# CAT-GxD: Centralized Access to Gene Expression Datasets

**DOI:** 10.1016/j.anaerobe.2025.103005

**Published:** 2025-10-13

**Authors:** Bryan Angelo P Roxas, Jennifer Lising Roxas, Jessica S. Guo, David S. LeBauer, Fiona McCarthy, Gayatri Vedantam, V.K. Viswanathan

**Affiliations:** 1 School of Animal and Comparative Biomedical Sciences, University of Arizona, Tucson, AZ; 2 Communications and Cyber Technology, University of Arizona, Tucson, AZ; 3 Department of Immunobiology, University of Arizona, Tucson AZ; 4 BIO5 Institute for Collaborative Research, University of Arizona, Tucson AZ; 5 Southern Arizona VA Healthcare System, Tucson AZ

**Keywords:** Proteomics, Transcriptomics, Multi-omic, *Clostridioides difficile*, Database, Bacterial Gene Expression Datasets

## Abstract

**Objectives:**

Extensive multi-omic bacterial gene expression datasets are publicly available, but tools that unify these datasets for interpretation and hypothesis-testing are limited. The complexity and requirement of specialized bioinformatics and programming expertise pose significant major barriers for researchers attempting to query and analyze these datasets. Our objective was to develop an integrated search engine that simplifies access to publicly available gene expression data to facilitate comparison and analysis of multi-omics datasets.

**Methods:**

We developed the Centralized Access to Gene Expression Datasets (CAT-GxD) search engine to provide integrated access to, and facilitate analysis of, publicly available transcriptomics and proteomics datasets of the CDC Urgent Threat pathogen *Clostridioides difficile*. Manual data curation was performed to integrate and standardize all 74 nonredundant transcriptomics and quantitative proteomics datasets available at Gene Expression Omnibus (GEO) database and ProteomeXchange Consortium. The CAT-GxD search engine, developed on open-source R-shiny framework, is available at https://viz.datascience.arizona.edu/catgxd/.

**Results:**

CAT-GxD successfully consolidated disparate transcriptomics and proteomics datasets, supporting interpretation and hypothesis testing. CAT-GxD provides customizable visualization of gene expression data under different conditions. We demonstrate the utility of CAT-GxD in analyzing the contribution of RNA polymerase, nitrogen-limitation N (RpoN) to *C. difficile* biology, and highlight the RpoN-dependent regulation of genes treated with succinate and the secondary bile acid deoxycholate.

**Conclusions:**

CAT-GxD streamlines the analysis of *C. difficile* multi-omic data, reducing the complexity and analysis time. It facilitates the generation of novel hypotheses and the identification of anti-infective targets, and can be adapted to incorporate data analysis paradigms for diverse organisms.

## INTRODUCTION

Publicly available gene expression compendia include transcriptomics (RNAseq and microarray) and proteomics datasets, as well as gene regulatory and protein structure information. For bacteria, such expression data are available for diverse isolates, mutant derivatives, and upon exposure to varied environmental conditions. While the datasets can be retrieved from the Gene Expression Omnibus (GEO) database [1] and ProteomeXchange Consortium [2], the data are not readily queryable and require biologists, who may have limited expertise in bioinformatics, to expend considerable time and effort to use the information. Integration of these individual datasets requires multiple analysis and curation steps to manage the use of non-standardized gene IDs across multiple datasets, compare results across different experimental trials (i.e., calculate replicate ratios), calculate fold change for each gene, and incorporate the constantly increasing number of dataset submissions. A resource whose main function is to compile and interrogate differential expression at the transcription and translation levels across different experiments supports both exploratory and analysis stages of a biologist’s research.

Initially focusing on the Centers for Disease Control (CDC) Urgent Threat pathogen *Clostridioides difficile,* we built CAT-GxD (Centralized Access to Gene Expression Datasets), an integrated and queryable engine to readily access and compare expression of specific genes or gene subsets in accordance with Findability, Accessibility, Interoperability, and Reusability (FAIR) guiding principles [3]. By providing seamless access to disparate public datasets and embedded tools for comparative analysis and discovery, CAT-GxD enables biologists to rapidly and effectively query public expression data to understand *C. difficile* gene expression and how it changes under different conditions.

The Gram-positive and spore-forming anaerobic pathogen *Clostridioides difficile* (formerly *Clostridium difficile)* is considered an ‘Urgent Threat’ to US healthcare by the CDC [4]. *C. difficile* is the leading cause of antibiotic-associated diarrhea that may be self-limiting, or progress to severe and fulminant (pseudomembranous) colitis, ileus or toxic megacolon [5]. Annual US cases of *C. difficile* infections (CDI) are approximately 500,000, with ~30,000 cases resulting in deaths, impose up to $6 billion overall cost to healthcare [6, 7]. There are currently no vaccines against CDI, and there are several limitations to antibiotic therapy, including the emergence of resistant strains and the perpetuation of gut dysbiosis. The mechanisms by which *C. difficile* causes disease are poorly understood and are an active area of investigation. The ~4,000 genes of *C. difficile* are controlled by complex regulatory networks that are responsive to metabolic and environmental cues; the functions of most of these genes in bacterial physiology and virulence remain undefined. Understanding their roles in pathogenesis will facilitate the development of novel therapeutic agents.

While *C. difficile* 630 has been widely used for various mechanistic studies, the genetic and phenotypic diversity, particularly of current clinical isolates, is well recognized. Indeed, publicly available transcriptomics and proteomics datasets have been generated with at least five different *C. difficile* strains, and cognate mutant derivatives. Here we introduce how we built CAT-GxD to access this larger data collection, and demonstrate the utility of this resource for users who wish to easily extract gene expression information from otherwise difficult-to-compare multi-omics datasets.

## METHODOLOGY

### Construction and Content

#### Implementation.

The CAT-GxD engine was developed on a R shiny (version 1.7.5) [8] framework that utilizes dplyr (version 1.1.4) [9] for data filtering, cyjShiny (version 1.0.42) [10] for interactive networks, heatmaply (version 1.5.0) [11] for interactive heatmap, ggbiplot (version 0.6.2) [12] for principal component analysis (PCA) visualization, and Pathview (version 1.46.0) [13] and KEGGREST (version 1.46.0) [14] for KEGG pathway analysis. The publicly accessible resource is published using the RStudio Connect platform, and 180 sample comparisons (referred to as ‘**RATIOS**’ to distinguish from numerical gene expression log2 ‘**ratios’**) derived from 74 omics datasets (Supplemental Table 1) are freely available at https://viz.datascience.arizona.edu/catgxd/. CAT-GxD has been validated on multiple web browsers including Mozilla Firefox, Google Chrome, Microsoft Edge, and Safari.

#### Data sets and Data Curation.

*C. difficile*-specific gene expression datasets and protein quantitation datasets were collected from the GEO [1] and ProteomeXchange [2] databases, respectively. We generated an SQL database using RSQlite (version 2.3.4) [15] to incorporate 63 nonredundant GEO transcriptomics datasets and 11 quantitative proteomic datasets listed in Supplemental Table 1. GEO subseries datasets already available in their respective superseries dataset were excluded. Proteomics datasets, focused on quantitative data were included while those focused on post-translational modification studies were excluded. Manual data curation was performed to integrate and standardize these datasets. Briefly, gene IDs were converted to the Ensembl stable IDs [16] for the *C. difficil*e 630 reference strain [17] using DAVID [18]. Homologs of the strains other than 630 were determined using BLASTp [19] and Artemis Comparison Tool (ACT) [20]. A gene was considered a homolog if the best BLASTp hit with >95% identity was in the same location as the 630 counterpart, and this was visually confirmed using ACT. Pre-computed log2 ratios without any gene duplication were imported as is. Studies with replicate log2 ratios assigned for a gene ID (common in microarray experiments due to replicate spots) were solved by computing the median log2 ratio. Two-channel microarray experiments with duplicate ratios assigned to a gene were solved by computing the average of the complementary ratios then log2 transformed. Reads Per Kilobase Million (RPKM) of raw RNASeq data were computed. For datasets without any computed ratios, log2 ratios were calculated for each treatment versus control, and median normalization was performed. Gene classifications (described below in *3.1 Input and Data Filtering*) were compiled to provide a comprehensive and robust search.

**Accessibility and Data Downloads.** Data analysis output can be downloaded from each of the analysis tabs. Output files are in the following formats: **.XLS** Excel files for the gene and study information tables,.**PNG** image files for the heatmap, principal component analysis plot, KEGG pathway analysis maps and STRING network analysis.

## RESULTS AND DISCUSSION

### A. Comparison of CAT-GXD to Existing Resources.

As of June 2025, publicly available datasets of *C. difficile* gene expression under different conditions include 93 transcriptomics datasets from the Gene Expression Omnibus (GEO) database [1] and 30 proteomics datasets from ProteomeXchange Consortium [2]. Of these datasets, all 74 nonredundant transcriptomics and quantitative proteomics datasets (Supplemental Table 1), as described in the methods, are accessible in CAT-GxD. Besides CAT-GxD, no other *C. difficile* proteomics analysis resource is currently available. However, the Bacterial and Viral Bioinformatics Research Center (BV-BRC) [21] and *C. difficile* Portal [22], are resources which aid in mining some of the *C. difficile* transcriptomics datasets.

BV-BRC was formed in 2019 under the National Institute of Allergy and Infectious Diseases (NIAID) Bioinformatics Resource Center (BRC) program. This resource provides access to multiple bioinformatic analysis tools to mine genomic and transcriptomics data of viral and bacterial pathogens including *C. difficile* [21]. BV-BRC (version 3.47.11) currently includes four *C. difficile* transcriptomics datasets, submitted to GEO database between 2010–2011.

The *C. difficile* Portal was launched in November 2021 and utilizes 11 transcriptomics datasets submitted to GEO database from 2010–2018. The goal of this resource is to predict regulatory and metabolic network models for *C. difficile* and assign functions to uncharacterized genes. These analyses are based upon two computational modeling algorithms, the environment and gene network (EGRIN) and the phenotype of regulatory influences integrated with metabolism and environment (PRIME) [22]. Both BV-BRC and *C. difficile* Portal focus on transcriptomics datasets derived from the *C. difficil*e strain 630 [17]. They do not integrate proteomic data, and are yet to be updated to include more recent public gene expression datasets.

### B. Using the CAT-GxD Resource.

CAT-GxD provides easy access to a large set of proteomics and transcriptomics datasets generated from multiple *C. difficile* strains and mutant derivatives publicly available as of June 2025, and captures gene expression changes in response to various environmental and growth conditions. We designed the user interface to allow researchers to easily navigate various analysis tools. An example with preset filters is included to help first-time users to navigate the CAT-GxD platform. The ‘Help’ tab links to a step-by-step guide on how the example analyses were performed.

#### Input and Data Filtering.

CAT-GxD accepts several search input types, including keywords and gene IDs (Figure 1). There are 3787 genes and 180 RATIOS or sample comparisons freely accessible. Entering a search term (keyword, gene name or gene ID) narrows the data frame to genes associated with the search term. In the Advanced Search tab, gene search parameters can be customized by selecting specific *C. difficile*-specific gene filters (amino acid synthesis, antibiotic, antibiotic resistance, bile, capsule, cell wall, conjugation, cortex, flagella, germination, iron metabolism, PaLoc, peptidoglycan, phage-associated, pili, quorum sensing, redox, replication, response regulator, secretion system, sensor kinase, sigma factors, spore coat, sporulation, sugar metabolism, surface layer proteins, toxin, transcription, transcription factor, and two-component systems), PSORTb (v3.0.3) [23] protein localization (cell wall, cytoplasmic, extracellular and membrane) [24], gene set (pangenome, core genome, metabolic core, and essential), and functional classification categories (enzyme class, clusters of orthologous genes (COG) [25], Kyoto Encyclopedia of Genes and Genomes (KEGG) Orthology [26], Rapid Annotation using Subsystem Technology (RAST) [27] Subsystems, UniProtKB [28] keywords, Gene Ontology [29] (GO) biological process, GO molecular function, and GO cellular component). Study filters can be customized by selecting specific omics type (proteomics and/or transcriptomics), category (e.g., antibacterial, antibiotic, bile acid, gene knockout, infection study, stress, and treatment), select experimental studies and specific RATIOS or sample comparisons.

#### Results and Data Visualization.

CAT-GxD presents the data as a gene information table, study information table, heatmap [11], principal component analysis (PCA) plot [12], KEGG pathway analysis maps [14], and STRING network analysis [30], accessible under separate tabs (Figure 1).

#### Gene Information Table.

The Gene Info tab provides the number of genes associated to the search term and a table containing the gene IDs, gene descriptions, isoelectric points (pI), molecular weights (MW), amino acid lengths, and PSORTb [23] localization. Gene IDs are hyperlinked to AlphaFold Protein Structure Database pages [31]. Gene descriptions are hyperlinked to NCBI Pubmed pages with pre-populated search words (gene name, gene ID, PMID) to aid in locating published papers describing the gene. Gene or operon coordinates are hyperlinked to National Center for Biotechnology (NCBI) Sequence Viewer 3.49.0 pages [32]. Columns can be sorted by clicking on the headers.

#### Study Information Table.

The study information table contains hyperlinks to specific GEO series accession number (GSExxx) [1], ProteomeXchange dataset identifier (PXD+six figure integer) [2], and publication associated with each RATIO or sample comparison. Specific samples compared in each RATIO are also listed in the numerator and denominator columns. Columns can be sorted by clicking on the headers.

#### Heatmap.

An interactive, hierarchically clustered heatmap presents log2 ratios for each gene (y-axis) plotted against selected RATIOS or sample comparisons (x-axis). Log2 ratio thresholds and heat map colors can be customized. Gene name, experiment ID and actual log2 ratio value can be visualized by hovering the cursor over a specific heatmap cell. Users can narrow the set of genes and experiments in view by dragging a rectangle around a desired region of the heatmap. Zoom and pan functions are also available via icons/buttons on the right, and above the heatmap. Increasing the figure height allows for separation of text that may be clustered on the X axis. Selecting the Cluster option displays the hierarchical clustering of genes and RATIOS.

#### Principal Component Analysis (PCA).

Genes with similar expression profiles can be easily visualized via the interactive PCA plot [12]. Users can select 2 or more RATIOS to be displayed on the PCA plot. Log2 ratio (‘stringency’) thresholds can also be customized. If desired, PCA plot can be zoomed into by dragging a rectangle around a region, or by using the buttons on the right, above the PCA plot.

#### Kyoto Encyclopedia of Genes and Genomes (KEGG) Pathway Analysis.

For each of the selected RATIO or sample comparison, gene expression log2 ratios are overlaid on selected KEGG analysis pathway maps [14]. Log2 ratio threshold can be customized. If Use Data Frame is selected, only log2 ratios of genes associated with the customized search will be overlaid on the selected KEGG Pathway maps. Clicking on the KEGG icon will transfer users to the KEGG site where the original pathway can be viewed along with the expression data.

#### Network.

For each of the selected RATIO or sample comparison, gene expression log2 ratios are overlaid on STRING networks [30]. STRING score thresholds can be customized. Users can also reposition nodes in the network. The output network is hyperlinked to the STRING Database page. Clicking on STRING will transfer users to the STRING site where the nature of interactions between the proteins can be gleaned (however, CAT-GxD expression data will not carry over).

### C. A Worked Example of how to use CAT-GXD.

The utility of CAT-GxD can be demonstrated by re-analyzing previously published data on the contribution of RpoN to *C. difficile* biology. RpoN (RNA polymerase, nitrogen-limitation N), also known as Sig54 (Sigma-54) or SigL, is an alternative sigma factor that facilitates bacterial adaptation to the environment. RpoN-binding promoter sites, and RpoN-dependent transcriptomic and proteomic changes in *C. difficile*, have been characterized by several groups including ours [33–35]. The following example of CAT-GxD utility was generated from the complete dataset available. Screen shots of the CAT-GxD portal are provided as Supplemental Figures 1–9, while a summary of the data analyses is depicted in Figures 2 and 3.

Using the Δ*rpoN* microarray dataset of Soutourina et al. as the reference RATIO [35], it was observed that of the 16 genes and 15 operons with predicted *rpoN* promoter sites, only 4 genes and 4 operons (totaling 28 genes, Supplemental Table 2) had reduced expression in Δ*rpoN* (last column) relative to the parent *C. difficile* 630 strain. Searching for these 28 genes (Supplemental Table 2 footnote) using the Quick Start search bar found in the CAT-GxD home page generated a heatmap wherein 156 of 180 RATIOS or sample comparisons had altered gene expression or protein levels by at least one-fold (Supplemental Figure 1).

Twenty of these RATIOS or sample comparisons exhibiting the most altered or divergent gene expression patterns (Supplemental Table 3), including the reference ‘Δ*rpoN*’ RATIO, were selected in the ‘Study Filters’ menu in the Advanced Search tab to reduce the size and complexity of the heatmap. The Heatmap (Figure 2A; Supplemental Figure 2) for these 20 conditions is clustered to highlight RATIOS with similar and dissimilar expression patterns relative to the ‘Δ*rpoN*’ RATIO. Three RATIOS - the reference (‘Δ*rpoN*’), the most similar (‘240 μM DCA 48 hrs’), and the most dissimilar (‘80 mM succinate 24 hr biofilm’) - were selected for more detailed analysis. Deoxycholate (DCA), a secondary bile acid, kills vegetative *C. difficile*, inhibits spore germination, reduces sporulation efficiency [36], and sub-lethal DCA concentrations stimulate biofilm formation [37]. Several gut bacteria, including *Bacteroides* spp., *Prevotella* spp. and *Firmicutes* spp., ferment dietary fibers and host-derived carbohydrates to produce succinate either as an intermediate metabolite or an end product [38]. Gut dysbiosis can lead to high levels of succinate, and succinate promotes biofilm formation by *C. difficile* [39].

When the ‘Δ*rpoN*’, ‘240 μM DCA 48 hrs’, and ‘80 mM succinate 24 hr biofilm’ RATIOS were selected under the PCA (Principal Component Analysis) tab, three gene clusters with similar expression patterns were identified (Figure 2B; Supplemental Figure 3A and B):

1. Cluster [1] - An anaerobic glutamate/electron transfer flavoprotein cluster (*hadA*, *hadI*, *hadB*, *hadC*, *acdB*, *etfB1*, and *etfA*1) predicted to belong to CD630_Operon0192,
2. Cluster [2] - A phosphotransferase system (PTS) cluster (CD0284, CD0285, CD0286, CD0287, CD0288, CD0289 and CD0290) predicted to belong to CD630_Operon0141 and sporulation membrane/peptidases (CD2697, CD2698 and CD2699) predicted to belong to CD630_Operon1727.
3. Cluster [3] - A butanoate metabolism (*buk2*, *iorB*, *iorA*, and CD2382) predicted to belong to CD630_Operon1528, pentose/glucuronate interconversion (CD2323 and CD2324) predicted to belong to CD630_Operon1495, and uronic acid metabolism (*kdgT1*, CD2871, and *uxaA*) predicted to belong to CD630_Operon1855.

Genes downregulated in the ‘Δ*rpoN*’ RATIO can also be viewed as a network of interactions defined by the STRING database network analysis [30]. The 26 genes resolved into six networks; CD1413 and CD3093 did not have interactions within the selected gene set (Figure 2C; Supplemental Figure 4).

Selecting specific RATIOS (for instance, ‘Δ*rpoN*’, ‘240 μM DCA 48 hrs’, and ‘80 mM succinate 24 hr biofilm’) on the pull-down tab will overlay gene expression data on the STRING network (**Figure C;** Supplemental Figures 4, 5, and 6); it should be emphasized that while all nodes in STRING represent proteins, the expression data within CAT-GxD may depict either mRNA or protein abundance based on the choice of filters. For the ‘Δ*rpoN*’ RATIO, the 26 genes are downregulated. In the ‘240 μM DCA 48 hrs’ RATIO, *rpoN* expression was decreased (log2 ratio= −2.576), and the downregulated expression of 18 genes (clusters [1] and [3]) mirrored the pattern observed in the ‘Δ*rpoN*’ RATIO. In contrast, *rpoN* upregulation (log2 ratio= 2.156) and increased expression of 13 genes in clusters [1] and [3] were observed in the ‘80 mM succinate 24 hr biofilm’ RATIO. This supports the hypothesis that RpoN levels impact the expression of Clusters [1] and [3] genes. Interestingly, 6 genes in cluster [2] showed similar downregulation patterns with succinate treatment and with RpoN loss. The similarity of cluster [2] gene expression between DCA and succinate treatment, respectively, despite the diverging *rpoN* levels suggests epistatic effects of the two treatments on RpoN-dependent regulation of this group of genes.

In the pathway tab in CAT-GxD, gene expression data for specific conditions can be overlaid on selected KEGG pathways. Five of the seven Phosphotransferase System (PTS) genes in cluster [2] are included in KEGG’s Phosphotransferase System (PTS) pathway (Figure 3; Supplemental Figures 7, 8, and 9). Four genes are involved in glucosaminate phosphorylation while CD0284 is involved in mannose phosphorylation. The PTS pathway is annotated with colored boxes highlighting sugar phosphorylation processes that distinguish the ‘Δ*rpoN*’, ‘240 μM DCA 48 hrs’, and ‘80 mM succinate 24 hr biofilm’ RATIOS. Most of the genes involved in Glc family phosphorylation and L-ascorbate phosphorylation were downregulated with both DCA and succinate treatments, unlike with Δ*rpoN* where they were unchanged. DCA differs from succinate exposure in increased expression of mannitol (*mtlF*) phosphorylation genes, and decreased sorbitol phosphorylation genes. Succinate- and DCA-exposure differed in their impacts on expression of genes involved in cellobiose, fructoselysine (a fructooligosaccharide) and sorbitol phosphorylation.

Both DCA and succinate promote biofilm formation by *C. difficile* with the latter inducing significantly thicker biofilms. The observed pathway differences may explain succinate’s ability to induce significantly thicker biofilms by *C. difficile*. Previous studies have linked increased cellobiose [39], fructooligosaccharides [40] and sorbitol [37] metabolism and phosphorylation to succinate-induced biofilm formation. Downregulation of *mtlF* (mannitol- specific phosphotransferase) involved in mannitol metabolism and phosphorylation has been observed in succinate-induced biofilms [39] but its levels have not been identified in DCA- or bile acid-induced biofilms. It can be hypothesized that the divergent regulation of *mtlF* underlies the differential impacts of DCA- and succinate-treatment on biofilm formation.

### D. Database Access and Update.

User access will be monitored via Google Analytics (https://analytics.google.com/analytics/web/). CAT-GxD will be updated periodically to include recently added proteomics and transcriptomics datasets at GEO) database [1] and ProteomeXchange Consortium [2]. Update release information can be found on the CAT-GxD ‘About’ tab.

Thus, CAT-GxD provides ready access to diverse *C. difficile* gene expression datasets, incorporates links to established analysis modules, and allows for new ways for visualizing the data to generate novel hypotheses.

## CONCLUSIONS

The scope and possibilities of informatics-driven bio-solutions are still meager compared to the scale of the global infectious disease burden. The lack of targeted (e.g., microbiota-sparing) anti-infective strategies over the past decade represents a huge unmet medical need with significant value proposition. CAT-GxD is an integrated platform enabling easy and customizable analysis of otherwise disparate bacterial pathogen gene expression datasets and, thereby, advancing our understanding of *C. difficile* pathogenesis and aid in the development of new therapeutic strategies.

## Supplementary Material

1 **Supplemental Table 1** is supplied as an Excel file.

2 **Supplemental Figure 1.** Heatmap data output from analyzing the 28 genes listed in Supplemental Table 2. Input term entered in the CAT-GxD’s Quick Start search bar is listed in the Supplemental Table 2 footnote. Heatmap colors were customized to green for ‘min’ and red for ‘max’, and Cluster option was selected.
**Supplemental Figure 2.** Heatmap data output when analysis of the 28 genes listed in Supplemental Table 2 was restricted to 20 RATIOS listed in Supplemental Table 3. To generate this heatmap, ‘Study Filters’ menu found in the Advanced Search tab was customized to perform ‘AND’ Logic analysis for the following selected RATIOS: ‘ΔcotL’, ‘500 ng/ml Myxopyronin B’, ‘spo0A-’, ‘5 mM cysteine 8 hrs’, ‘2% O2’, ‘ΔclnR 2 ug/ml LL-37’, ‘Δfur 0 μM iron-sulfate’, ‘Δfur 15 μM iron-sulfate’, ‘ΔrpoN’, ‘ΔsigB 0 ng/ul anhydrotetracyclin’, ‘ΔhfQ’, ‘240 μM DCA 9 hrs’, ‘240 μM DCA 24 hrs’, ‘240 μM DCA 48 hrs’, ‘80 mM succinate 24hr biofilm’, ‘ΔCD1687 240uM DCA’, ‘ATC induced CD1687’, ‘*E. faecalis* coculture’, ‘10 mM trehalose’, ‘630 nutrient shift’.
**Supplemental Figure 3. (A)** Principal Component Analysis (PCA) plot output when the following RATIOS were analyzed: ‘Δ*rpoN’*, ‘240 μM DCA 48 hrs’, and ‘80 mM succinate 24 hr biofilm’. **(B)** Gene Info output for the 28 genes listed in Supplemental Table 2. Predicted CD operons and Operon location or gene coordinates are shown.
**Supplemental Figure 4.** STRING network output when ‘Δ*rpoN’* RATIO was analyzed using 28 genes listed in Supplemental Table 2. The 28-gene input term listed in the Supplemental Table 2 footnote was entered in the CAT-GxD’s ‘Search’ bar located in the Advanced Search tab. 26 of the initial 28 genes resolved into clusters.
**Supplemental Figure 5.** STRING network output when ‘240 μM DCA 48 hrs’ RATIO was analyzed using 28 genes listed in Supplemental Table 2. The 28-gene input term listed in the Supplemental Table 2 footnote was entered in the CAT-GxD’s ‘Search’ bar located in the Advanced Search tab.
**Supplemental Figure 6.** STRING network output when ‘80 mM succinate 24 hr biofilm’ RATIO was analyzed using 28 genes listed in Supplemental Table 2. The 28-gene input term listed in the Supplemental Table 2 footnote was entered in the CAT-GxD’s ‘Search’ bar located in the Advanced Search tab.
**Supplemental Figure 7.** KEGG Pathway output for ‘Δ*rpoN*’ RATIO displaying ‘Phosphotransferase System (PTS)’ pathway. ‘Use Data Frame’ was not selected to overlay all identified genes in the ‘Δ*rpoN*’ RATIO in the selected pathway.
**Supplemental Figure 8.** KEGG Pathway output for ‘240 μM DCA 48 hrs’ RATIO showing Phosphotransferase System (PTS) pathway. ‘Use Data Frame’ was not selected to overlay all identified genes in the ‘240 μM DCA 48 hrs’ RATIO in the selected pathway.
**Supplemental Figure 9.** KEGG Pathway output for ‘80 mM succinate 24 hr biofilm’ RATIO showing Phosphotransferase System (PTS) pathway. ‘Use Data Frame’ was not selected to overlay all identified genes in the ‘80 μM succinate 24 hrs biofilm’ RATIO in the selected pathway.
**Supplemental Table 2.** Gene coordinates of the 28 genes with reduced expression in Δ*rpoN* relative to the parent strain (Soutourina et al microarray dataset [35]).
**Supplemental Table 3.** Selected RATIOS used in the example

## Figures and Tables

**Figure 1.**
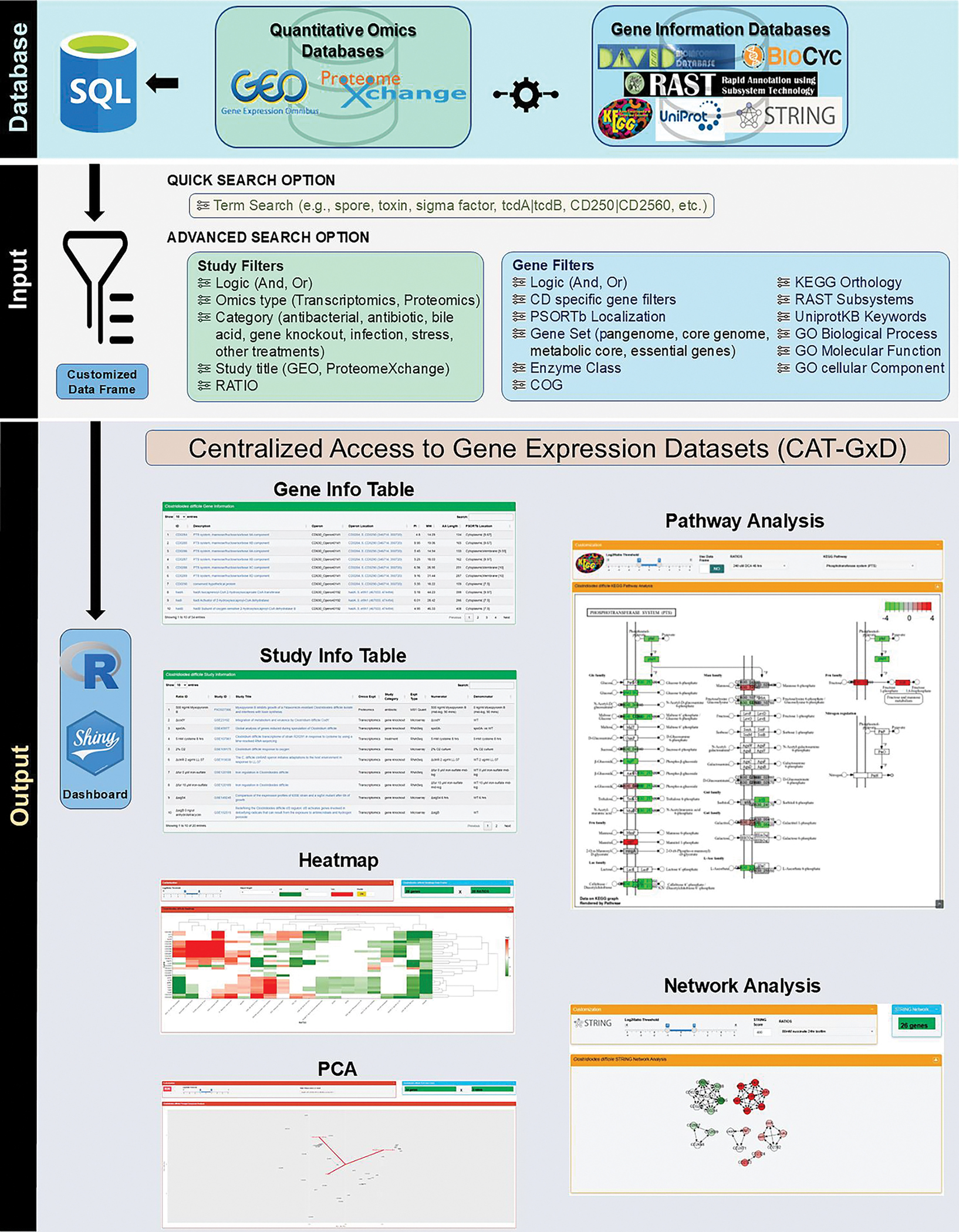
Sample CAT-GxD workflow. Quantitative omics datasets for CDC Urgent Threat pathogen *C. difficile* available from GEO and ProteomeXchange databases were curated and integrated, along with gene information from Gene ontology, KEGG, COG, RAST and STRING databases [1, 2, 25, 26, 27, 29, 30], to build CAT-GxD search engine. Users can select preferred experimental and gene filters to create a customized data frame. Gene expression data can be visualized as gene information table, study information table, heatmaps, PCA plots, STRING network analysis and KEGG pathway maps [11, 12, 14, 30].

**Figure 2.**
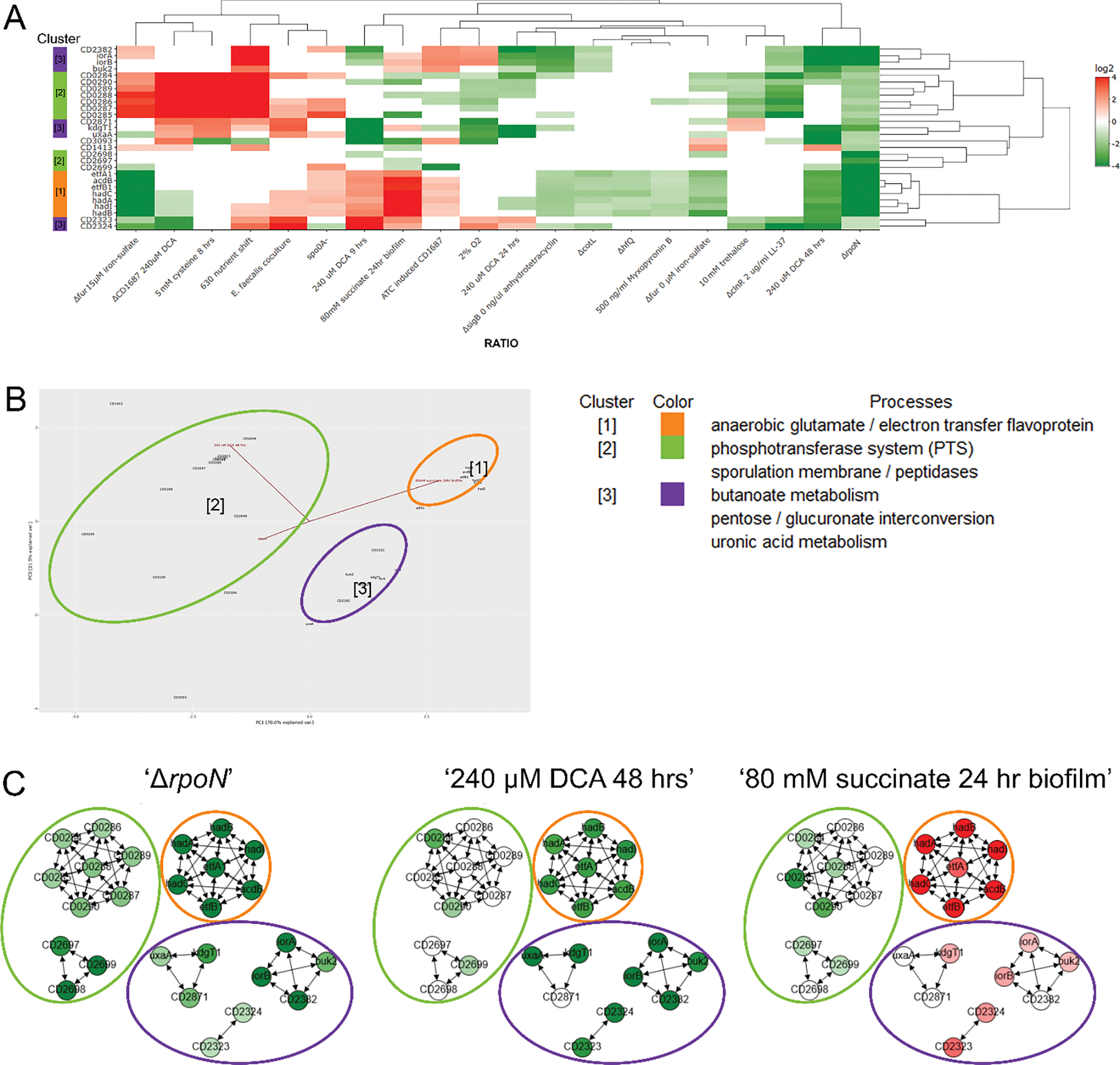
Example: Clustering and network analysis of genes and operons regulated by RpoN. (A) Heatmap analysis shows *C. difficile* exposed to deoxycholic acid (‘240 μM DCA 48 hrs’ RATIO) and succinate (‘80 mM succinate 24 hr biofilm’ RATIO) visually produced the most similar and dissimilar gene expression profiles to *rpoN* deletion (‘Δ*rpoN*’ RATIO), respectively. (B) Principal component analysis (PCA) of the 28 genes against the 3 selected RATIOS (‘Δ*rpoN*’, ‘240 μM DCA 48 hrs’, and ‘80 mM succinate 24 hr biofilm’) identifies 3 gene clusters with similar gene expression patterns. (C) STRING database analysis defined interactions result in 5 separate networks. A more granular analysis of *rpoN* regulation is achieved by overlaying gene expression data onto the network.

**Figure 3.**
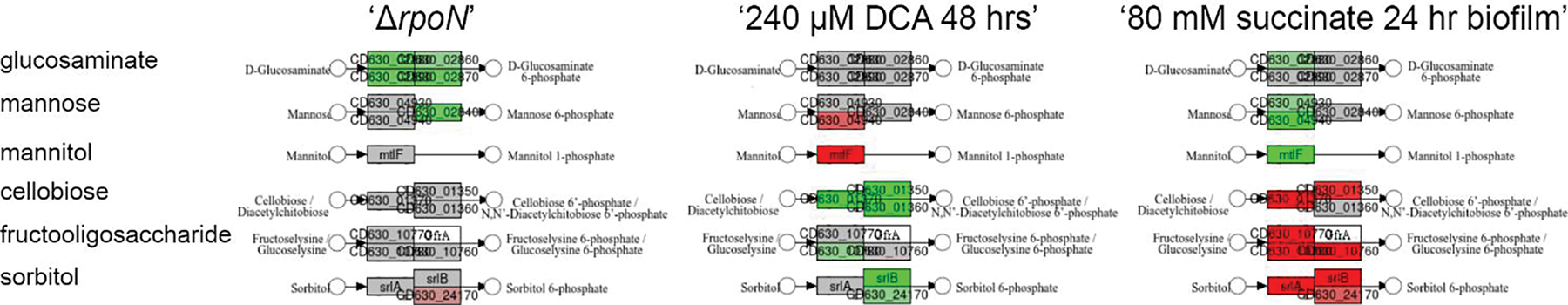
Example: Pathway analysis of differentially regulated sugar phosphotransferase system (PTS). The PTS was identified in network analysis to not be directly affected by *rpoN* levels (Figure 2C). CAT- GxD’s pathway analysis was used to determine differences in PTS gene regulation when not limited to set of genes regulated by RpoN. Upregulated cellobiose, fructooligosaccharide and sorbitol PTS genes observed in the analysis have been shown in previous studies to contribute to a significantly thicker biofilm when induced by succinate [37, 39, 40].

## Data Availability

The CAT-GxD platform can be accessed at viz.datascience.arizona.edu/catgxd/. Details about the datasets analyzed in CAT-GxD are provided in Supplemental Table 1. The datasets are accessible via NCBI GEO (https://www.ncbi.nlm.nih.gov/geo/) and ProteomeXchange (https://proteomecentral.proteomexchange.org/cgi/GetDataset).

## Highlights

- CAT-GxD provides integrated access to public *C. difficile* gene expression data
- CAT-GxD is a queryable engine that aids in analyzing disparate omics datasets
- CAT-GxD enables rapid query of gene expression changes under various conditions

## ABBREVIATIONS:

CAT-GxD: Centralized Access to Gene Expression Datasets
CDC: Center for Disease Control and Prevention
FAIR: Findability, Accessibility, Interoperability, and Reusability
CDI: *Clostridioides difficile* infection
GEO: Gene Expression Omnibus
BV-BRC: Bacterial and Viral Bioinformatics Research Center
NIAID: National Institute of Allergy and Infectious Diseases
BRC: Bioinformatics Resource Center
EGRIN: Environment and Gene Network
PRIME: phenotype of regulatory influences integrated with metabolism and environment
PCA: principal component analysis
RPKM: Reads Per Kilobase Million
KEGG: Kyoto Encyclopedia of Genes and Genomes
RAST: Rapid Annotation using Subsystem Technology
GO: Gene Ontology
pI: isoelectric point
MW: molecular weight
NCBI: National Center for Biotechnology
