# Supplementary material for: CAT-GxD: Centralized Access to Gene Expression Datasets
Source: Anaerobe. Author manuscript; Available in PMC 2026 Oct 13. (PMC12704217; doi:10.1016/j.anaerobe.2025.103005)
Supplement: Supplementary file 1 [file NIHMS2118613-supplement-2.docx]

**Supplemental Table 1** is supplied as an Excel file.

**Supplemental Table 2.** Gene coordinates of the 28 genes with reduced expression in Δ*rpoN* relative to the parent strain (Soutourina et al microarray dataset [35]).

| **Gene Coordinates** | **Gene ID** | **Gene Description** |
| --- | --- | --- |
| CD630_02840 | CD0284 | PTS system, mannose/fructose/sorbose IIA component |
| CD630_02850 | CD0285 | PTS system, mannose/fructose/sorbose IIB component |
| CD630_02860 | CD0286 | PTS system, mannose/fructose/sorbose IIA component |
| CD630_02870 | CD0287 | PTS system, mannose/fructose/sorbose IIB component |
| CD630_02880 | CD0288 | PTS system, mannose/fructose/sorbose IIC component |
| CD630_02890 | CD0289 | PTS system, mannose/fructose/sorbose IID component |
| CD630_02900 | CD0290 | conserved hypothetical protein |
| CD630_03950 | *hadA* | hadA Isocaprenoyl-CoA:2-hydroxyisocaproate CoA-transferase |
| CD630_03960 | *hadI* | *hadI* Activator of 2-hydroxyisocaproyl-CoA dehydratase |
| CD630_03970 | *hadB* | hadB Subunit of oxygen-sensitive 2-hydroxyisocaproyl-CoA dehydratase B |
| CD630_03980 | *hadC* | hadC Subunit of oxygen-sensitive 2-hydroxyisocaproyl-CoA dehydratase C |
| CD630_03990 | *acdB* | acdB Acyl-CoA dehydrogenase, short-chain specific |
| CD630_04000 | *etfB1* | etfB1 Beta-subunit of electron transfer flavoprotein |
| CD630_04010 | *etfA1* | etfA1 Alpha-subunit of electron transfer flavoprotein |
| CD630_14130 | CD1413 | putative membrane protein |
| CD630_23230 | CD2323 | putative sugar-phosphate dehydrogenase |
| CD630_23240 | CD2324 | putative sugar-phosphate dehydrogenase |
| CD630_23790 | *buk2* | buk2 Butyrate kinase (Branched-chain carboxylic acid kinase) |
| CD630_23800 | *iorB* | iorB Indole pyruvate ferredoxin/flavodoxin oxidoreductase |
| CD630_23810 | *iorA* | iorA Indole pyruvate ferredoxin/flavodoxin oxidoreductase |
| CD630_23820 | CD2382 | putative pyridoxal phosphate-dependent transferase |
| CD630_26970 | CD2697 | putative peptidase, M20D family |
| CD630_26980 | CD2698 | putative membrane protein |
| CD630_26990 | CD2699 | putative membrane protein |
| CD630_28700 | *kdgT1* | kdgT1 2-keto-3-deoxygluconate permease 1 |
| CD630_28710 | CD2871 | uxaA' putative N-terminus region of carbohydrate hydrolase, SAF domain |
| CD630_28720 | *uxaA* | uxaA D-galactate dehydratase/Altronate hydrolase |
| CD630_30930 | CD3093 | putative gamma-glutamyl-gamma-aminobutyrate hydrolase |

**Footnote:**

28-gene input term

CD630_02840|CD630_02850|CD630_02860|CD630_02870|CD630_02880|CD630_02890|CD630_02900|CD630_03950|CD630_03960|CD630_03970|CD630_03980|CD630_03990|CD630_04000|CD630_04010|CD630_14130|CD630_23230|CD630_23240|CD630_23790|CD630_23800|CD630_23810|CD630_23820|CD630_26970|CD630_26980|CD630_26990|CD630_28700|CD630_28710|CD630_28720|CD630_30930

**Supplemental Table 3.** Selected RATIOS used in the example

| **RATIO** | **Study Title** |
| --- | --- |
| Δ*cotL* | CotL, a new morphogenetic coat protein of *Clostridium* *difficile* |
| 500 ng/ml Myxopyronin B | Myxopyronin B inhibits growth of a Fidaxomicin-resistant *Clostridioides* *difficile* isolate and interferes with toxin synthesis |
| spo0A- | Global analysis of genes induced during sporulation of *Clostridium* *difficile* |
| 5 mM cysteine 8 hrs | *Clostridium* *difficile* transcriptome of strain R20291 in response to cysteine by using a time-resolved RNA-sequencing |
| 2% O2 | *Clostridium* *difficile* response to oxygen |
| ΔclnR 2 ug/ml LL-37 | The *C.* *difficile* *clnRAB* operon initiates adaptations to the host environment in response to LL-37 |
| Δfur 0 μM iron-sulfate | Iron regulation in *Clostridioides* *difficile* |
| Δfur 15 μM iron-sulfate | Iron regulation in *Clostridioides* *difficile* |
| Δ*rpoN* | Comparison of the expression profiles of 630E strain and a sig54 mutant after 6h of growth |
| Δ*sigB* 0 ng/ul anhydrotetracyclin | Redefining the *Clostridioides* *difficile* σB regulon: σB activates genes involved in detoxifying radicals that can result from the exposure to antimicrobials and hydrogen peroxide |
| Δ*hfQ* | An RNA-centric global view of *Clostridioides* *difficile* reveals broad activity of Hfq in a clinically important Gram-positive bacterium |
| 240 uM DCA 9 hrs | Metabolic adaption to extracellular pyruvate triggers biofilm formation in *Clostridioides* *difficile* |
| 240 uM DCA 24 hrs | Metabolic adaption to extracellular pyruvate triggers biofilm formation in *Clostridioides* *difficile* |
| 240 uM DCA 48 hrs | Metabolic adaption to extracellular pyruvate triggers biofilm formation in *Clostridioides* *difficile* |
| 80mM succinate 24hr biofilm | Extracellular succinate induces spatially organized biofilm formation in *Clostridioides* *difficile* |
| ΔCD1687 240uM DCA | The cell wall lipoprotein CD1687 acts as a DNA binding protein during deoxycholate-induced biofilm formation in *Clostridioides* *difficile* |
| ATC induced CD1687 | The cell wall lipoprotein CD1687 acts as a DNA binding protein during deoxycholate-induced biofilm formation in *Clostridioides* *difficile* |
| *E. faecalis* coculture | Enterococci enhance *Clostridioides* *difficile* pathogenesis |
| 10 mM trehalose | Transcriptomic RNA-sequencing analysis of the hypervirulent *Clostridioides* *difficile* R20291 strain in response to trehalose |
| 630 nutrient shift | Differential stress transcriptome landscape of historic and recently emerged hypervirulent strains of *Clostridium* *difficile* strains determined using RNA-seq |

**Supplemental Figure 1.** Heatmap data output from analyzing the 28 genes listed in Supplemental Table 2. Input term entered in the CAT-GxD’s Quick Start search bar is listed in the Supplemental Table 2 footnote. Heatmap colors were customized to green for ‘min’ and red for ‘max’, and Cluster option was selected.

**Supplemental Figure 2.** Heatmap data output when analysis of the 28 genes listed in Supplemental Table 2 was restricted to 20 RATIOS listed in Supplemental Table 3. To generate this heatmap, ‘Study Filters’ menu found in the Advanced Search tab was customized to perform ‘AND’ Logic analysis for the following selected RATIOS: ‘ΔcotL’, ‘500 ng/ml Myxopyronin B’, ‘spo0A-‘, ‘5 mM cysteine 8 hrs’, ‘2% O2’, ‘ΔclnR 2 ug/ml LL-37’, ‘Δfur 0 μM iron-sulfate’, ‘Δfur 15 μM iron-sulfate’, ‘ΔrpoN’, ‘ΔsigB 0 ng/ul anhydrotetracyclin’, ‘ΔhfQ’, ‘240 μM DCA 9 hrs’, ‘240 μM DCA 24 hrs’, ‘240 μM DCA 48 hrs’, ’80 mM succinate 24hr biofilm’, ‘ΔCD1687 240uM DCA’, ‘ATC induced CD1687’, ‘*E. faecalis* coculture’, ‘10 mM trehalose’, ‘630 nutrient shift’.

**Supplemental Figure 3. (A)** Principal Component Analysis (PCA) plot output when the following RATIOS were analyzed: ‘Δ*rpoN’*, ‘240 μM DCA 48 hrs’, and ‘80 mM succinate 24 hr biofilm’. **(B)** Gene Info output for the 28 genes listed in Supplemental Table 2. Predicted CD operons and Operon location or gene coordinates are shown.

**Supplemental Figure 4.** STRING network output when ‘Δ*rpoN’* RATIO was analyzed using 28 genes listed in Supplemental Table 2. The 28-gene input term listed in the Supplemental Table 2 footnote was entered in the CAT-GxD’s ‘Search’ bar located in the Advanced Search tab. 26 of the initial 28 genes resolved into clusters.

**Supplemental Figure 5.** STRING network output when ‘240 μM DCA 48 hrs’ RATIO was analyzed using 28 genes listed in Supplemental Table 2. The 28-gene input term listed in the Supplemental Table 2 footnote was entered in the CAT-GxD’s ‘Search’ bar located in the Advanced Search tab.

**Supplemental Figure 6.** STRING network output when ‘80 mM succinate 24 hr biofilm’ RATIO was analyzed using 28 genes listed in Supplemental Table 2. The 28-gene input term listed in the Supplemental Table 2 footnote was entered in the CAT-GxD’s ‘Search’ bar located in the Advanced Search tab.

**Supplemental Figure 7.** KEGG Pathway output for ‘Δ*rpoN*’ RATIO displaying ‘Phosphotransferase System (PTS)’ pathway. ‘Use Data Frame’ was not selected to overlay all identified genes in the ‘Δ*rpoN*’ RATIO in the selected pathway.

**Supplemental Figure 8.** KEGG Pathway output for ‘240 μM DCA 48 hrs’ RATIO showing Phosphotransferase System (PTS) pathway. ‘Use Data Frame’ was not selected to overlay all identified genes in the ‘240 μM DCA 48 hrs’ RATIO in the selected pathway.

**Supplemental Figure 9.** KEGG Pathway output for ‘80 mM succinate 24 hr biofilm’ RATIO showing Phosphotransferase System (PTS) pathway. ‘Use Data Frame’ was not selected to overlay all identified genes in the ‘80 μM succinate 24 hrs biofilm’ RATIO in the selected pathway.
